# Prevalence of Autism Spectrum Disorders and the Years Lived With Disability in Eastern Sub‐Saharan Africa, From 1990 to 2023

**DOI:** 10.1002/brb3.71663

**Published:** 2026-08-11

**Authors:** Sebsibe Tadesse, Boko Loka Safayi, Abdulbasit Sherfa, Musa Jemal, Begetayinoral Kussia Lahole, Melese Chego Cheme, Sewhareg Belay, Asqual Gebreslassie Gebremariam, Gemechis Tuke, Abinet Zewudie, Gelgelo Wodessa, Geleta Nenko Dube

**Affiliations:** ^1^ School of Public Health, Institute of Health Bule Hora University Bule Hora Ethiopia; ^2^ National Data Management and Analytics Center for Health, Ethiopian Public Health Institute Addis Ababa Ethiopia; ^3^ Department of Pediatrics and Child Health nursing, Institute of Health Bule Hora University Bule Hora Ethiopia; ^4^ Department of Public Health, College of Medicine and Health Science Werabe University Werabe Ethiopia; ^5^ Department of Midwifery, College of Medicine and Health Sciences Arba Minch University Arba Minch Ethiopia; ^6^ School of Public health, Institute of Health Sciences Wollega University Wollega Ethiopia; ^7^ College of Medicine and Health Sciences Hawassa University Hawassa Ethiopia; ^8^ Faculty of Public Health, College of Health Sciences Mekelle University Mekelle Ethiopia; ^9^ Department of psychiatry, School of Nursing and Midwifery, Institute of Health Bule Hora University Bule Hora Ethiopia

**Keywords:** autism spectrum disorders, Eastern Sub‐Saharan Africa, Global Burden of Diseases study, prevalence, YLDs

## Abstract

**Background:**

Autism spectrum disorders are lifelong neurodevelopmental conditions affecting social interaction, communication, and behavior. Despite rising global burden, data from Eastern Sub‐Saharan African region are limited. This study estimated the prevalence of ASD and the years lived with disability in the region from 1990 to 2023.

**Methods:**

We analyzed input data from the Global Burden of Diseases 2023 study for the ESSA countries. Prevalence and years lived with disability were estimated using disease modeling with Bayesian meta‐regression and spatiotemporal Gaussian process regression. Age‐standardized rates were calculated, and 95% uncertainty intervals (UIs) were generated. Trends were assessed for statistical significance based on UIs.

**Results:**

In 2023, an estimated 4.24 million individuals (95% UI: 2.02, 8.80) were living with ASD in the ESSA region, with an age‐standardized prevalence of 814 per 100,000 population (95% UI: 405, 1481). Autism spectrum disorders contributed 0.8 million YLDs (95% UI: 0.3, 1.9), and the age‐standardized YLDs rate was 154 per 100,000 population (95% UI: 70, 333). Prevalence and YLDs showed no significant sex or location differences and remained nearly unchanged from 1990 to 2023.

**Conclusion:**

The study indicated that ASD is a significant and persistent health burden in the ESSA region, affecting young children and adolescents disproportionately. There is an urgent need for early detection of cases, strengthening collaborative initiatives, and implementing culturally sensitive, community‐based interventions to support individuals with ASD and their caregivers. Moreover, improved surveillance and standardized diagnostic practices are important to guide policy and resource allocation.

## Graphical abstract

Using Global Burden of Disease 2023 data, a regional analysis of Eastern Sub‐Saharan Africa from 1990–2023 found that 4.24 million people lived with autism spectrum disorders, accounting for 0.81 million years lived with disability. Stable trends underscore the need for earlier diagnosis, standardized diagnosis and culturally appropriate interventions.

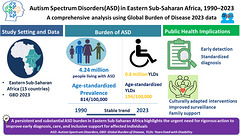

## 1 Introduction

Autism spectrum disorders (ASD) are a diverse group of conditions in which individuals experience challenges with social interaction and communication, along with unusual or repetitive behaviors and activity patterns (WHO 2025). Worldwide, an estimated 1 in 127 people were living with ASD in 2021, according to the Global Burden of Diseases (GBD) 2021 estimates (Santomauro et al. 2025). However, prevalence estimates vary widely across regions, countries and studies. For instance, in the United States, the prevalence of ASD among 8‐year‐old children in 2022 was 32.2 per 1000, or approximately one in 31 children. Evidence from low‐ and middle‐income regions further highlights variability in estimates. A systematic review from the Middle East and North Africa region found that ASD prevalence varies widely depending on diagnostic methods and sampling approaches, with uncertain trends over time (Akomolafe et al. 2025). In contrast, epidemiological data from the Eastern Sub‐Saharan Africa (ESSA) region remain limited and fragmented due to limited diagnostic capacity, and weak reporting systems (Shaw et al. 2025; Aderinto et al. 2023; Adams 2024). This highlights a critical gap in understanding the true burden of ASD in the region. Using GBD 2023, this study provides comparable ASD prevalence and disability estimates to address the gaps and inform decision‐making.

Although ASD cannot be prevented, early detection and timely intervention are critical to improve its outcomes. Public health approaches emphasis early screening during well‐child visits to enable timely diagnosis and access to interventions that improve development and quality of life (Francis et al. 2021; Qin et al. 2024). Several community‐level screening tools, including the Modified Checklist for Autism in Toddlers (M‐CHAT) and the Social Communication Questionnaire (SCQ), are widely used in high‐income settings. However, they may not fully capture cultural and social nuances in non‐Western populations, limiting their applicability in settings like the ESSA region (Alasiri et al. 2024; Marvin et al. 2017). Clinical techniques include therapies and early intervention programs that address social and behavioral skills, and communication issues using concepts from developmental and behavioral approaches (Zhuang et al. 2024; Okoye et al. 2023). Moreover, evidence‐based interventions such as the Early Start Denver Model (ESDM) and Applied Behavior Analysis (ABA) have shown improvements in cognitive, communication, and behavioral outcomes in high‐income settings. However, their high costs, reliance on trained professionals, and resource requirements limit their accessibility in low‐resource settings (Alasiri et al. 2024; Kumar and Bhattacharya 2024).

In low‐resource settings evidence‐based and context‐appropriate interventions for ASD include caregiver‐mediated and parent‐training programs, which improve social communication and adaptive functioning (Bhat 2024; Whitehouse et al. 2021). Task‐sharing approaches using non‐specialist health workers help expand access to structured developmental support (Naithani et al. 2022). Adapted school‐based inclusive education and simplified behavioral interventions further enhance feasibility and sustainability in resource‐limited contexts (World Health Organization 2023).

Generating complete, comparable, and robust epidemiological evidence on ASD burden across demographic and geographic groups over time is challenging, particularly in resource‐constrained settings. To address this gap, this study estimated the prevalence and years lived with disability attributable to ASD in the ESSA region from 1990 to 2023 using the GBD 2023 framework. The study findings may contribute for informed decision‐making, foster inter‐country collaborations, targeted resource allocation, and track progress in meeting targets.

## 2 Methods and Materials

### 2.1 Study Setting

This study was done in the ESSA region, which housed 15 countries; namely, Somalia, Malawi, Burundi, Comoros, Djibouti, Eritrea, Madagascar, Mozambique, Tanzania, Rwanda, Ethiopia, South Sudan, Uganda, Kenya, and Zambia. In the region, an estimated 482 million people lived in 2024. More than two‐threes, 68.5%, of the population resided in rural areas, where there is only limited access to mental healthcare services (Carroll 2012). Given the scarcity of reliable population‐based data and weak surveillance systems in such settings, the GBD 2023 framework is appropriate as it generates standardized, comparable estimates using model‐based approaches across countries and time.

### 2.2 Data Sources

The study used input data from the GBD 2023 study's data repository, the global health data network. The complete list of the data sources is found at: https://ghdx.healthdata.org/gbd‐2023/sources.

### 2.3 Data Analyses

Disease modeling with Bayesian meta‐regression (DisMod‐MR 2.1) was used to estimate the non‐fatal health outcomes (i.e., prevalence and years lived with disability (YLDs)) of autism spectrum disorder. The 95% uncertainty intervals (UIs) were derived from the 2.5th and 97.5th percentiles of 1000 posterior draws. Spatiotemporal Gaussian process regression (ST‐GPR) was applied to smooth variation in trend. Demographic variations were adjusted by age‐standardizing the rate. Percentage change between 1990 and 2023 was declared significant when the 95% UI did not contain the zero percent change.

### 2.4 Ethical Considerations

This study was conducted as part of the GBD Collaborators Network and in adherence to the GBD Protocol. The study used secondary data from publicly available sources and did not require individual informed consent. The study adhered to established data privacy standards and ethical guidelines governing the use of secondary health data, ensuring confidentiality and responsible use of all included data sources.

## 3 Results

### 3.1 Prevalence

**FIGURE 1:**
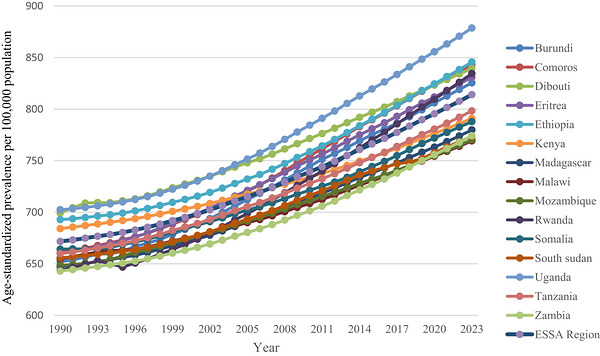
Trends of age‐standardized prevalence rate of autism spectrum disorders in ESSA region, 1990 to 2023. The estimates represent modeled average values, and their trends were not statistically significant when considered with their 95% UIs.

**FIGURE 2:**
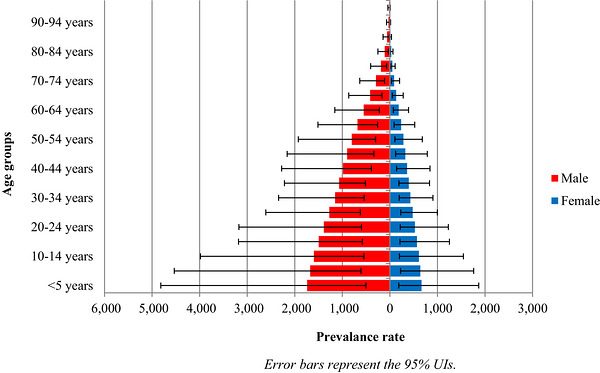
Age‐sex specific distribution of the prevalence rate of autism spectrum disorders in ESSA region, 2023.

There were an estimated 4.2 million population (95% UI: 2.0, 8.8) living with ASD in the ESSA region in 2023, comprising 3.1 million males (95% UI: 1.5, 6.4) and 1.2 million females (95% UI: 0.6, 2.4). This was equivalent to an age‐standardized prevalence rate of 814 cases per 100,000 population (95% UI: 405, 1481), with 1189 cases per 100,000 males (95% UI: 596, 2162) and 445 cases per 100,000 females (95% UI: 219, 817). There was no significant difference in the prevalence rate by sex and location across the region, as the estimated numerical differences were not supported by non‐overlapping 95% uncertainty intervals. Between 1990 and 2023, the age‐standardized prevalence rate remained nearly unchanged, likely due to limited changes in diagnostic capacity and consistent methodological assumptions in GBD modeling over time (Table 1 and Figures 1 and 2)

**TABLE 1:** Prevalence of autism spectrum disorder and YLDs by sex and location in the ESSA region, 1990 to 2023.

Location	Sex	Prevalence			YLDs		
		Number, 2023	Age‐standardized rate, 2023	% change, 1990–2023	Number, 2023	Age‐standardized rate, 2023	% change, 1990–2023
ESSA region	Male	3,069,572 (1,475,421, 6,364,870)	1189 (596, 2162)	0.21 (–0.41, 1.73)	585,977 (247,319, 1,360,044)	225 (104, 483)	0.23 (–0.41, 1.76)
	Female	1,165,654 (546,552, 2,442,462)	445 (219, 817)	0.21 (–0.42, 1.80)	221,251 (91,688, 524,932)	84 (37, 183)	0.23 (–0.43, 1.87)
	Both	4,235,226 (2,021,973, 8,801,875)	814 (405, 1481)	0.21 (–0.41, 1.76)	807,228 (339,007, 1,888,280)	154 (70, 333)	0.23 (–0.41, 1.80)
Burundi	Male	340,976 (194,063, 611,858)	1199 (611, 2148)	0.24 (–0.40, 1.78)	19,126 (7839, 44,525)	242 (128, 431)	0.25 (–0.40, 1.78)
	Female	133,285 (71,417, 249,500)	447 (224, 822)	0.25 (–0.40, 1.86)	7178 (2973, 16,862)	93 (46, 176)	0.26 (–0.40, 1.86)
	Both	474,261 (265,551, 861,358)	826 (419, 1481)	0.27 (–0.39, 1.84)	26,303 (10,842, 61,088)	166 (87, 302)	0.27 (–0.38, 1.83)
Comoros	Male	216,966 (103,849, 468,407)	1220 (620, 2171)	0.26 (–0.38, 1.79)	1134 (492, 2514)	227 (103, 489)	0.28 (–0.38, 1.87)
	Female	86,391 (40,668, 185,950)	456 (226, 830)	0.26 (–0.39, 1.87)	409 (173, 933)	84 (37, 182)	0.27 (–0.39, 1.95)
	Both	303,357 (144,899, 653,755)	843 (425, 1512)	0.28 (–0.37, 1.84)	1543 (671, 3453)	156 (72, 335)	0.29 (–0.37, 1.92)
Djibouti	Male	153,339 (72,952, 334,952)	1181 (604, 2094)	0.19 (–0.41, 1.62)	1825 (831, 3927)	215 (99, 447)	0.20 (–0.41, 1.74)
	Female	53,965 (25,090, 120,181)	441 (221, 797)	0.19 (–0.42, 1.66)	584 (254, 1267)	80 (35, 174)	0.20 (–0.40, 1.81)
	Both	207,304 (98,042, 454,884)	839 (428, 1495)	0.20 (–0.41, 1.65)	2409 (1088, 5210)	146 (66, 310)	0.22 (–0.41, 1.75)
Eritrea	Male	132,349 (64,022, 273,920)	1193 (611, 2151)	0.24 (–0.40, 1.76)	9305 (4096, 20,779)	232 (106, 483)	0.27 (–0.38, 1.88)
	Female	51,855 (24,567, 108,013)	444 (224, 822)	0.24 (–0.41, 1.84)	3242 (1413, 7420)	86 (38, 186)	0.27 (–0.41, 1.91)
	Both	184,203 (88,589, 381,529)	831 (424, 1507)	0.26 (–0.39, 1.81)	12,547 (5482, 28,261)	160 (73, 339)	0.29 (–0.38, 1.94)
Ethiopia	Male	804,063 (355,079, 1,767,691)	1229 (564, 2377)	0.22 (–0.42, 1.94)	153,810 (59,561, 380,859)	225 (105, 467)	0.24 (–0.43, 1.97)
	Female	297,977 (129,767, 654,552)	458 (208, 894)	0.22 (–0.43, 2.04)	56,701 (21,913, 142,153)	83 (37, 176)	0.24 (–0.44, 2.13)
	Both	1,102,040 (484,846, 2,421,467)	846 (386, 1637)	0.22 (–0.42, 1.98)	210,511 (81,385, 522,866)	159 (73, 334)	0.24 (–0.43, 2.01)
Kenya	Male	94,360 (46,214, 189,346)	1155 (530, 2267)	0.15 (–0.44, 1.75)	62,961 (25,212, 149,932)	233 (99, 536)	0.17 (–0.44, 1.77)
	Female	36,323 (17,590, 72,940)	431 (196, 849)	0.16 (–0.45, 1.83)	23,611 (9395, 56,621)	86 (36, 202)	0.17 (–0.46, 1.89)
	Both	130,682 (63,819, 262,023)	791 (361, 1552)	0.16 (–0.44, 1.79)	86,572 (34,538, 206,553)	160 (68, 371)	0.17 (–0.45, 1.82)
Madagascar	Male	100,295 (48,427, 209,295)	1142 (582, 2034)	0.21 (–0.41, 1.69)	38,346 (16,181, 86,632)	226 (102, 476)	0.23 (–0.41, 1.77)
	Female	37,765 (17,735, 81,075)	426 (214, 778)	0.21 (–0.42, 1.75)	14,590 (6063, 34,066)	84 (38, 181)	0.23 (–0.41, 1.98)
	Both	138,059 (66,312, 290,085)	780 (395, 1403)	0.20 (–0.42, 1.70)	52,935 (22,280, 120,300)	157 (71, 330)	0.22 (–0.41, 1.76)
Mozambique	Male	9563 (4815, 17,824)	1134 (581, 1999)	0.18 (–0.42, 1.60)	25,298 (10,945, 57,654)	219 (93, 493)	0.19 (–0.43, 1.70)
	Female	3080 (1516, 5805)	423 (213, 765)	0.18 (–0.43, 1.66)	9843 (4192, 22,618)	81 (34, 186)	0.19 (–0.42, 1.81)
	Both	12,643 (6335, 23,606)	769 (391, 1370)	0.17 (–0.42, 1.61)	35,141 (15,195, 80,235)	150 (63, 340)	0.19 (–0.42, 1.68)
Malawi	Male	137,994 (65,839, 293,356)	1143 (587, 2038)	0.18 (–0.43, 1.63)	41,241 (17,310, 96,204)	215 (100, 443)	0.20 (–0.42, 1.69)
	Female	53,189 (25,018, 112,102)	426 (215, 779)	0.18 (–0.43, 1.69)	16,333 (6686, 39,317)	80 (37, 170)	0.19 (–0.44, 1.85)
	Both	191,183 (90,884, 405,105)	770 (392, 1393)	0.19 (–0.43, 1.67)	57,575 (23,952, 135,665)	146 (67, 304)	0.21 (–0.42, 1.76)
Rwanda	Male	200,301 (98,035, 412,935)	1228 (625, 2196)	0.29 (–0.37, 1.88)	18,041 (7767, 40,495)	217 (102, 452)	0.31 (–0.38, 1.94)
	Female	76,677 (36,646, 158,885)	458 (228, 842)	0.30 (–0.37, 1.97)	6920 (2990, 15,695)	80 (35, 174)	0.32 (–0.36, 2.15)
	Both	276,978 (134,750, 571,095)	835 (421, 1514)	0.30 (–0.38, 1.92)	24,961 (10,771, 56,376)	148 (69, 312)	0.32 (–0.38, 1.95)
Somalia	Male	329,259 (148,388, 686,211)	1136 (583, 2070)	0.20 (–0.44, 1.73)	29,388 (12,427, 70,036)	215 (99, 453)	0.21 (–0.43, 1.75)
	Female	124,274 (55,224, 258,740)	422 (212, 796)	0.19 (–0.44, 1.78)	10,243 (4080, 25,409)	80 (37, 171)	0.21 (–0.44, 1.88)
	Both	453,533 (203,611, 944,896)	788 (402, 1454)	0.19 (–0.45, 1.73)	39,631 (16,860, 95,445)	145 (67, 308)	0.20 (–0.43, 1.76)
South Sudan	Male	48,834 (24,209, 97,745)	1128 (575, 2031)	0.21 (–0.41, 1.73)	12,218 (5166, 28,825)	233 (104, 491)	0.23 (–0.40, 1.73)
	Female	17,065 (8286, 35,001)	420 (211, 776)	0.21 (–0.43, 1.79)	4493 (1825, 10,480)	87 (39, 186)	0.23 (–0.41, 1.88)
	Both	65,899 (32,513, 132,598)	774 (391, 1415)	0.18 (–0.43, 1.69)	16,711 (6979, 39,305)	159 (72, 337)	0.19 (–0.43, 1.71)
Uganda	Male	428,302 (207,745, 885,861)	1278 (764, 2130)	0.24 (–0.35, 1.75)	65,062 (33,161, 130,878)	216 (103, 454)	0.27 (–0.35, 1.79)
	Female	166,896 (79,599, 346,095)	492 (277, 837)	0.27 (–0.37, 1.87)	25,345 (12,041, 52,253)	79 (35, 174)	0.30 (–0.38, 2.10)
	Both	595,198 (288,270, 1,230,776)	879 (515, 1471)	0.25 (–0.36, 1.78)	90,407 (45,372, 184,017)	149 (70, 319)	0.28 (–0.36, 1.87)
Tanzania	Male	5941 (2947, 11,716)	1174 (600, 2074)	0.20 (–0.40, 1.66)	81,516 (35,040, 185,600)	222 (104, 461)	0.23 (–0.40, 1.69)
	Female	2159 (1047, 4295)	439 (219, 791)	0.20 (–0.41, 1.71)	31,528 (13,716, 73,605)	82 (37, 174)	0.22 (–0.43, 1.90)
	Both	8100 (4002, 15,995)	798 (405, 1420)	0.21 (–0.40, 1.68)	113,044 (48,865, 260,873)	151 (70, 316)	0.23 (–0.41, 1.73)
Zambia	Male	64,497 (31,156, 136,626)	1138 (580, 2019)	0.20 (–0.41, 1.67)	26,223 (11,127, 61,335)	212 (97, 450)	0.22 (–0.41, 1.75)
	Female	23,791 (11,353, 49,666)	425 (212, 774)	0.20 (–0.42, 1.74)	10,049 (4098, 24,150)	79 (35, 171)	0.22 (–0.42, 1.94)
	Both	88,288 (42,521, 186,276)	774 (391, 1393)	0.20 (–0.41, 1.70)	36,271 (15,373, 85,330)	145 (65, 313)	0.22 (–0.42, 1.76)

### 3.2 Years Lived With Disability

**FIGURE 3:**
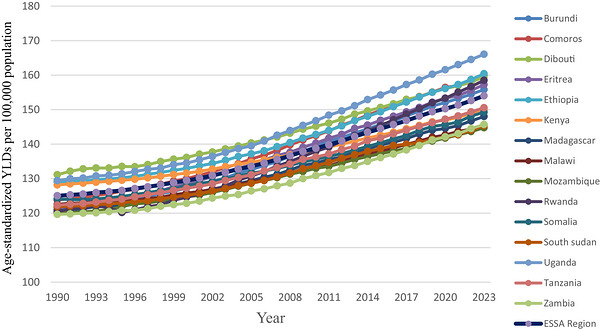
Trends of age‐standardized YLDs rate from autism spectrum disorders in the ESSA region, 1990 to 2023. The estimates represent modeled average values, and their trends were not statistically significant when considered with their 95% UIs.

**FIGURE 4:**
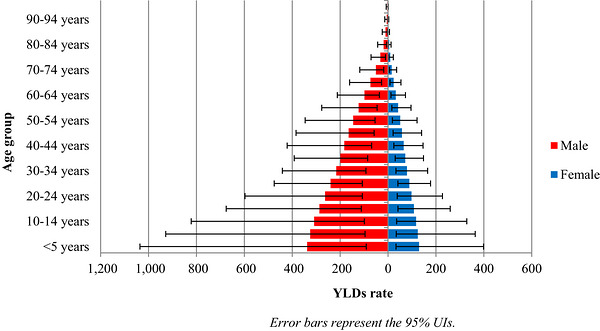
Age‐sex specific distribution of the YLDs rate from autism spectrum disorders in the ESSA region, 2023.

In 2023, there were an estimated 0.8 million YLDs (95% UI: 0.3, 1.9) attributable to ASD in the ESSA region, with 0.6 million YLDs among males (95% UI: 0.3, 1.4) and 0.2 million among females (95% UI: 0.1, 0.5). The age‐standardized YLDs rate were 154 per 100,000 population (95% UI: 70, 333), with 225 per 100,000 males (95% UI: 104, 483) and 84 per 100,000 females (95% UI: 37, 183). No significant sex and location differences were estimated in the YLDs rate. Compared to the rate in 1990, the age‐standardized YLDs rate remained nearly unchanged in 2023 (Table 1 and Figures 3 and 4).

## 4 Discussion

This study aimed to estimate the prevalence and YLDs attributable to ASD in the ESSA region from 1990 to 2023 using the standardized methods of the GBD 2023 estimate. The findings indicate that ASD affects a significant number of individuals in the region, with an estimated 4.24 million prevalent cases and over 0.8 million YLDs in 2023. Both the age‐standardized prevalence and YLDs rates have remained stable over time, likely reflecting GBD modeling methods that rely on limited data and apply smoothing across locations and years (Santomauro et al. 2025).

The age‐standardized prevalence rate of ASD was estimated at 814 cases per 100,000 populations in the ESSA region in 2023, representing about 1 in 123 individuals living with ASD. These estimates are consistent with the previous global GBD estimates in 2021 (Santomauro et al. 2025). Nonetheless, the prevalence of ASD in the ESSA region remains markedly lower than that reported in high‐income countries. For instance, in the United States, the estimated prevalence in 2022 was 32 per 1000 almost equivalent to 1 in every 31 children (Shaw et al. 2025). The lower estimated prevalence in ESSA region is likely due to limited data, cultural stigma, and restricted access to diagnostic services and trained specialists; rather than true differences in incidence (Aderinto et al. 2023; Bamford et al. 2025). Cultural perceptions of neurodevelopmental conditions, stigma, and reliance on non‐medical explanatory models may further reduce care‐seeking and case identification (Durkin et al. 2015; Franz et al. 2017). In addition, the use of diagnostic tools developed in high‐income settings, which may not fully capture local cultural and behavioral contexts, can contribute to underreporting (Alasiri et al. 2024; Marvin et al. 2017).

The lack of significant variation across countries further reflects widespread gaps in detection and reporting across the region (Bamford et al. 2025; World Health Organization 2023). However, these should be interpreted cautiously. While point estimates differ, overlapping uncertainty intervals suggest no clear differences; however, this may also reflect uniform data limitations and modeling assumptions across countries. Similarly, the estimated YLDs rate indicates substantial disability associated with ASD, consistent with global evidence that ASD contributes significantly to long‐term functional and social impairment (Santomauro et al. 2025).

Age‐specific patterns show the highest prevalence among young children and adolescents, underscoring the importance of early recognition and timely support for individuals with ASD and their caregivers. Lower prevalence estimates among adults likely reflect historical under diagnosis, limited adult diagnostic services, and the absence of developmental screening systems in earlier decades (Shaw et al. 2025; Ebrahimi Meimand et al. 2023; Kantawala et al. 2023). These findings reinforce the need for life‐course approaches to ASD care, including improved transition services into adulthood.

The stable ASD prevalence and YLDs rate estimated between 1990 and 2023 may reflect limited progress in improving detection, reporting systems, and service availability in the region rather than true epidemiological stability. Moreover, the stagnant trends in the region suggest ongoing gaps in ASD diagnostic capacity and insufficient prioritization of neurodevelopmental conditions within national health systems. In contrast, several high‐income countries have reported rising ASD prevalence due to increased awareness, expanded diagnostic criteria, and improved early identification (Shaw et al. 2025; Elsabbagh et al. 2012).

The findings underscore the need for integrated policies to strengthen early detection and support systems across the region. Integrating neurodevelopmental screening into routine child health services and educational policies could improve early identification. Expanding training for primary care providers, teachers, and community health workers would enhance diagnostic and referral capacity. In addition, public awareness initiatives may reduce stigma and promote service use (Bamford et al. 2025). Moreover, investing in collaborative initiatives, culturally adapted, behavioral interventions, and caregiver training is essential to mitigate long‐term disabilities.

The study has limitations inherent to GBD data. GBD estimates rely on multiple data sources with varying quality, particularly in low‐resource settings. This reliance on limited primary data and modeling assumptions may underestimate the true burden and smooth local variation (Santomauro et al. 2025). Despite these limitations, the study provides the most comprehensive and comparable estimates of ASD burden in the region to date, offering a foundation for policy and programmatic planning. Strengthening population‐based surveillance, investing in epidemiological studies, and standardizing diagnostic practices across countries are essential to improve data quality. Regional collaboration and increased funding for neurodevelopmental health services are strongly recommended.

## 5 Conclusion

Autism spectrum disorders remain a substantial and persistent health burden in the ESSA region, with stable age‐standardized prevalence and YLDs rates from 1990 to 2023. The comparatively low prevalence relative to high‐income settings probably reflects under diagnosis and limited diagnostic capacity.

Improving ASD outcomes in the region requires strengthening early detection through integration into primary healthcare, expanding diagnostic capacity via task‐sharing and implementing culturally appropriate community‐based interventions. Future efforts should prioritize strengthening surveillance systems, evaluating context‐specific intervention models, and generating high‐quality local data to better inform policy and resource allocation.

## Author Contributions

Melese Chego Cheme: formal analysis, visualization, Writing – review and editing. **Boko Loka Safayi**: investigation, formal analysis, visualization, writing – review and editing, writing – original draft. **Abdulbasit Sherfa**: formal analysis, visualization, writing – review and editing. **Begetayinoral Kussia Lahole**: formal analysis, visualization, writing – review and editing. **Abinet Zewudie**: formal analysis, visualization, writing – review and editing. **Geleta Nenko Dube**: visualization, writing – review and editing, formal analysis. **Sebsibe Tadesse**: conceptualization, writing – original draft, writing – review and editing, methodology, supervision. **Musa Jemal**: formal analysis, visualization, writing – review and editing. **Asqual Gebreslassie Gebremariam**: formal analysis, visualization, writing – review and editing. **Sewhareg Belay**: formal analysis, visualization, writing – review and editing. **Gemechis Tuke**: formal analysis, visualization, writing – review and editing. **Gelgelo Wodessa**: formal analysis, visualization, writing – review and editing.

## Funding

The authors have nothing to report.

## Ethics Statement

Ethical approval was not required for the present study involving humans because the data included were publicly available from the GBD Data Exchange database. All methods in this study were performed according to the Guidelines for Accurate and Transparent Health Estimates Reporting (GATHER).

## Conflicts of Interest

The authors declare no conflicts of interest.

## Data Availability

The data we used in this article are publicly available online on the official website of the Institute of Health Metrics and Evaluation (https://collab2023.healthdata.org/gbd‐results/).
